# Exploring first grade medical students’ professional identity using metaphors: implications for medical curricula

**DOI:** 10.3402/meo.v19.20876

**Published:** 2014-02-13

**Authors:** Hunkar Korkmaz, Yesim Y. Senol

**Affiliations:** 1 Faculty of Education, Division of Curriculum & Instruction, Hacettepe University, Ankara, Turkey; 2 Faculty of Medicine, Medical Education, Akdeniz University, Antalya, Turkey

**Keywords:** medical curriculum, professional identity, metaphors, medical students

## Abstract

**Background:**

Although professional identity development is an important concept in medical education, the process has not been well-investigated from a student perspective.

**Purpose:**

This study examines the metaphorical images formulated by first grade medical students in Turkey to describe physicians in the context of establishing a professional identity, along with its limitations.

**Method:**

Participants (*N=*148) completed the prompt: *A physician is like* _____ *because* _____ to indicate their conceptualizations of physician. The data were analyzed both qualitatively and quantitatively.

**Results:**

Altogether, 71 well-articulated metaphorical images were identified – comprising six conceptual themes.

**Conclusions:**

While subject to some limitations, the use of metaphors to formulate and describe professional identities can be helpful in reflecting the personal beliefs and values of matriculants to medical school, as well as providing some guidance and feedback to curriculum development efforts.

## Introduction

Until the late 1970s, there was little discussion of the need to add ‘professionalism’ and ‘personal values within a humanist context’ to the medical curriculum (1, 2). Since then, educators and organizations like the Association of American Medical Colleges (AAMC) and the Accreditation Council for Graduate Medical Education (ACGME) have developed innovative curricular experiences to expose students to the issues of professionalism and to promote knowledge of ethical principles related to personal values (3), skills of moral reasoning (4), and the development of humanistic attitudes. However, little is known about the developmental processes through which a medical student elaborates his or her personal experiences during their training, and about the way he or she gradually develops a professional identity.

As Reisetter and associates state: ‘Professional identity is the view of ones self as a professional, plus competence as a professional, resulting in congruence between personal worldview and professional view’ (5). Indeed, studies examining medical students’ professional identity development from a social constructionist perspective have shown how medical students develop an understanding about the boundaries of their profession, and the ways in which they may interact with others as part of an interprofessional healthcare team. These sets of beliefs, attitudes, and understanding about their roles, within the context of work, generally refer to their ‘professional identity’ (6–9).

Thus, the way we perceive ourselves influences our choice of action and judgment. Thus, understanding medical students’ professional identity is important in gaining insight into the essential aspects of medical students’ future professional lives, such as their career decision making, motivation, job satisfaction, emotion, and commitment. Moreover, existing studies across various cultures have considered physicians’ professional identity as a key factor in physicians’ motivation, effectiveness, and retention through qualitative research (1, 10–15).

Reports of research in which a metaphor was employed as a tool for analysis exist across a wide range of medical subjects including medicine (16), education of pathology (17), immunology (18), mental health (19, 20), primary health (21), and HIV prevention (22). Researchers, drawing on the work of Lakoff and Johnson (23), describe how beginners used personal metaphors to integrate the past, present, and future to help them find cohesion within their lives. The power of the metaphor, they contend, is in its ability to ‘clarify meaning in the midst of complexity’ (23) – making it a useful research tool in understanding the individual physician in terms of particular social contexts.

Within the medical education context, the act of reflecting on medical metaphors enables students to assemble a more reasoned perspective on the reality of professional health care service. In an annual AAMC meeting document, the teaching of communication skills among medical students was highlighted through the use of metaphors (24). Metaphors can also help medical students articulate and ‘construct representations of themselves and their experience to promote awareness of professional practice’ (25, 26). From this point of view, examining a variety of metaphors pertaining to medical education, we might be able to gain a good understanding of how medical students in different socio-cultural contexts see themselves, their patients, their work, their health care (i.e., What is it like to be a physician?). From a curricular standpoint, students’ expectations and behaviors – if defined early in the process – can help construct or organize the learning environment accordingly.

Within this aforementioned framework, this study examines the use of metaphors among Turkish medical students to answer the following questions: (1) What metaphorical images do Turkish first grade medical students use to describe a physician? (2) What conceptual themes can be derived from these metaphorical images? and (3) What implications can be derived for medical curricula and further research?

## Materials and methods

In Turkey, prospective students who: (1) have completed a 12-year primary and secondary education; (2) are in the top 1.5% of achievement levels; and (3) perform adequately on the national Students Selection and Placement Center (OSYM) exam are eligible for university medical training. Undergraduate medical education normally lasts 6 years, but can extend an additional year due to English preparatory programs in some medical faculties.

In late 2012, data were collected in Turkish using a structured, self-administered questionnaire containing the prompt: *A physician is like* _____ *because* _____. The study population consisted of all first-year medical students enrolled in the Faculty of Medicine, Akdeniz University (Turkey). Prospective participants were informed about the nature of the study, and permission to use (anonymously) material from their essays was obtained. In terms of data collection, a blank piece of paper containing the aforementioned prompt was distributed with the simple instruction to complete it by focusing on a single metaphorical image. Additionally, several close-ended demographic questions were included at the bottom of the page. The congruence between the ‘metaphor topic’ (i.e., physician) and the ‘metaphor vehicle’ (i.e., source domain) was emphasized through the use of the words ‘like’ and ‘because’. By writing about a metaphorical image that represented their professional thinking, participants were expected to make their implicit beliefs explicit. The participants were given 15 min to complete this task, since we wanted to elicit immediate reactions to the ‘physician’ prompt rather than exhaustive essays on the topic.

The data analysis followed the methodology of metaphor analysis. According to Moser (27), metaphor analysis is essentially a qualitative research methodology related to content analysis – but which also allows researchers to apply quantitative procedures to the categorical data that emerge from the underlying meanings and reasoning proffered by participants in each metaphorical relationship. We followed the data analysis stages and actions set forth by Saban, Kocbeker, and Saban (28): (1) naming and labeling; (2) sorting (clarification and elimination); (3) deciding the unit of analysis; (4) sample metaphor compilation and categorization; (5) establishing inter-rater reliability; and (6) quantitative data analysis.

In brief, all of the metaphorical images supplied by the participants were reviewed independently by the two authors. In the first stage, all metaphorical images were initially coded (e.g., river, machine, robot, etc.), and papers in which no metaphorical image was clearly articulated were eliminated. For example, some participants simply wrote nothing at all – leaving the paper blank. Others provided their views of certain characteristics rather than introducing a recognizable metaphorical image (e.g., ‘A physician is an active person in the medical process’). Others, while mentioning a metaphor, could not provide a rationale for their reasoning (e.g., ‘A physician is like an animal because _____’). Finally, some metaphors were so idiosyncratic that they could not be placed under a clearly recognizable theme (e.g., ‘A physician is like a leukocyte because s/he is always silent in the hospital’). For these reasons, a total of 84 papers were eliminated from the study.

All written phrases and expressions that either expressed or implied medical students’ understanding of a physician were marked. These marked passages were then compared by the two authors and coded according to a framework inspired by Johnson's schemata-based structures of understanding (29). The 71 coded metaphors were then organized aphetically and scrutinized (in order) to identify a sample ‘exemplar’ that best represented each of the identified metaphorical images. The next stage was to extract from the 71 surface metaphors the generative categories (i.e., conceptual themes) that they represented. Using inductive content analysis, six major conceptual categories were identified. Inter-coder reliability of the qualitative data was assessed using Miles and Huberman's (30) formula (Reliability=Agreement/Agreement+Disagreement×100). Reliability was also assessed through the use of the intra-class correlation coefficient (ICC), which provides an estimate of the level of rater agreement as well as rater consistency. Lastly, we calculated counts (*n*) and percentages (%) of metaphors in each category.

## Results

Study subjects included 298 medical students enrolled in the Faculty of Medicine, Akdeniz University in Turkey – representing approximately 72% of eligible participants (see ‘Coding and Elimination Stage’ above). Overall, males constituted 55.6% of the analyzable sample, and participants averaged 18.9 years of age (SD – 1.2). For individual items, the ICC ranged from 0.71 to 0.99; ICC for the total scores was 0.98. The results show a high degree of agreement among rates in scoring the items in the balance scale.

Based on quantitative results (counts and percentages) of the open-ended responses, a total of 148 study participants produced 71 identifiable ‘physician’ metaphors – which, in turn, were situated within six underlying conceptual themes, including: (1) the physician as a figure of kindness and help; (2) the physician as a superior figure; (3) the physician as a knowledge provider; (4) the physician as a health care provider; (5) the physician as a cooperative leader; and (6) the physician as a symbol of prestige.

### The physician as a figure of kindness and help

A figure of ‘kindness and help’ was the most prevalent physician image. Altogether, 59 participants (39.9%) and 14 metaphors (19.7%) constituted this conceptual theme – with dominant themes being *mother* (15), *friend* (15), *father* (8), *parents* (7), and *consultant* (4). The main characteristics of this category of metaphors include the following statements:

- *A physician is like **a mother** because she is **compassionate** and **ready to help at any time***.
- *A physician is like **a friend** because he/she is ready to **support all the time***.
- *A physician is like **a father**. He is **confidential and entrusting in his life***.
- *A physician is like **a parent** because he/she always **accepts you unconditionally***.
- *A physician is like **a consultant** because he/she is **ready to help at any time***.
- *A physician is like **an angel** because he/she **helps everyone and solves all problems***.

Other metaphorical images relating to this theme were a/an: helper, brother or sister, chocolate, coffee, wall, emotional woman, humanist, one of us.

### The physician as a superior figure – idealist and authoritative

Altogether, this conceptual theme was expressed by 31 students (20.9%) and in 15 (21.1%) of the metaphors – the most common being *artist* (5), *supernatural power* (4), *savior* (4), *magician/wizard* (2), *genius* (2), *House, MD* (2), and *ant* (2). The main characteristics of this category of metaphors include the following:

- *A physician is like **an artist** because he/she can **shape the patients’ body and recreate a new person using esthetic surgery methods***.
- *A physician is like a **supernatural power** because he/she can **give a new life***.
- *A physician is like a **robot** because he/she must **work non-stop and unconditionally***.
- *A physician is like **House, MD** because he/she has the **power to find a cure for incurable illnesses***.
- *A physician is like **a magician (wizard)** because he/she can **achieve difficult and unbelievable things***.
- *A physician is like **an ant** because he/she works very hard while the others enjoy themselves*.

Within this conceptual theme, other metaphorical images included a/an: soldier, race horse, robot, machine, stone, detective, paleography expert, judge, captive, and caterpillar.

### The physician as a knowledge provider

In total, 26 students (16.8%) and 15 (21.1%) metaphors comprised this conceptual theme, with the metaphors of *sun* (7), *teacher* (3), and *candle* (2) being most common. The main characteristics of this category of metaphors include:

- *A physician is like **a candle** and is like **a sun** that **lights the minds***.
- *A physician is a like **a teacher** because he/she **dispenses knowledge to patients and society** every day*.

Other metaphorical images relating to this knowledge provider theme were light, Google, Bing, Wikipedia, God,encyclopedia, book, evolution, fire, wise, full moon, computer, and newspaper.

### The physician as a health provider

Sixteen students (10.8%) and 11 metaphors (15.5%) constituted this conceptual theme, with the metaphors of *water* (5) and *medicine* (2) being the most dominant. The main characteristics of this category included:

- *The physician is like **water** because water is essential for the human body. A physician is necessary and **life: the heart's blood** for human health, like water*.
- *
A physician is like a **medicine** because he can **cure and save lives***.
- *A physician is like **a melon** because he/she is **like a medicine** for hot summer days*.
- *A physician is like **a healing distributor** because he/she **provides health service** and **dispense health to his/her patients and society***.
- *A physician is **a breath** because **life would be without him/her***.

Other metaphorical images included soil, a leukocyte, a detergent, a curer, and a river.

### The physician as a cooperative leader

A total of 12 students (8.1%) and 11 metaphors (15.5%) constituted this conceptual theme, with *musician–orchestra–conductor* (2) being most prevalent. In this theme, the other metaphorical images are mirror, directional signal, the earth, lion, tree, lighthouse, and coach. This category of metaphors includes the following main characteristics:

- *A physician is like **a musician** (orchestra conductor) because he/she is **active in participation and cooperation for public health***.
- *A physician is like **Mustafa Kemal Atatürk** (the founder and first minister of the Republic of Turkey) because he/she **works together in harmony (with others) for public health and is a leader of health reform***.
- *A physician like is **the pole star** because he/she **shows the direction***.
- *A physician like is **a rainbow** because he/she **hugs everyone from the sky***.

### The physician as a symbol of prestige

A total of five students (3.8%) students and five (7.0%) metaphors constituted this final conceptual theme, with the metaphors of a/an *aged wine* (1), *diamond* (1), *antique* (1), *gold mine* (1), and *handmade wall clock* (1) being among those mentioned. The characteristics of this category of metaphors include the following:

- *A physician is like **a gold mine** because he/she has **a long and difficult machining process***.
- *A physician is **an aged wine** because he/she is a valuable and very important person*.

## Discussion/conclusion

This study explored first grade medical students’ perceptions of the professions through the use of metaphors which, first and foremost, offered important insights into the structure of the contemporary Turkish medical education system. The qualitative analysis of the participant-generated metaphors showed that the students generally had positive perceptions of a physician. For example, two of the Turkish students’ most prevalent images were ‘kindness and help’ (39.9%) and ‘superior: idealist and authoritative’ (20.9%). These findings further suggest that many physician candidates believe that they have professional duties to health-related issues outside their direct clinical practices, and that these are considered to be individual responsibilities, as expressed through community participation. These findings are consistent with a view of professionalism in which physicians have a responsibility to contribute to helping the society that grants them professional status (31–33).

Roughly one-fifth (20.9%) of participants defined a physician as a superior, idealist, and authoritative figure. Here, it may be that the learning environment influences students’ perceptions and behavior. For instance, Akdeniz University Hospital, where participants were educated, has one of the largest and most experienced transplant practices in Turkey (and the world) – with highly skilled professionals in more than a dozen specialties working together to ensure quality patient care. In 2011, a Turkish surgical team successfully performed the country's first-ever full-face transplant and the world's first womb transplant from a cadaveric donor. Other significant transplants involving tissue, eyes, corneas, hearts, heart valves, bone marrow, etc. have all put Akdeniz University Hospital in the national and international spotlight.

Such operations rekindle hope for those suffering incurable illnesses. Consequently, certain students may have perceived a physician as an artist, a supernatural power, a savior, a magician–wizard, a genius, and House, MD under the aura of these remarkable developments. Similarly, other participants within this conceptual theme defined a physician as a race horse, a robot, a machine, a captive, and a caterpillar. There may be ample reason for this result: the Turkish health care system has recently been going through a series of crucial reforms in which the performance-based pay system has skewed incentives toward hospital-based employment and physician specialization.

Although not directly reflecting an impact on our findings, the potential effect of the mass media can perhaps be discussed relative to students’ metaphorical images. Two participants defined a physician as House, MD, a fictional television character, in their metaphorical image of a physician, suggesting that some students may derive their meanings about medicine from informal learning experiences that include portrayals in mass media. Medical educators can make use of these experiences to engage and involve a wide range of students by inviting them to share their thoughts and questions about television's medical-themed stories. Nevertheless, to realize Bybee's (34) recommendation that the efforts of both formal and free-choice education sectors be combined to move toward the goal of achieving science literacy for all, further exploration of the effects of specific involvement of television science in the classroom is needed.

First grade medical students’ third metaphor of physician was as a ‘knowledge provider’ (16.8%). To be eligible for medical school in the Turkish education system, a student must attain among the highest scores in the university entrance exam. Thus, in Turkish society, medical students are seen as an elite group in terms of mental abilities and knowledge even before they become physicians. On the contrary, the perception of a physician as knowledge provider may be associated with kindness and help: physicians should help their patients by providing knowledge or answering the patients’ questions about their illness and other health problems. Patients trust physicians with their health and, indeed, with their lives.

The remaining three metaphorical concepts of a physician were as ‘health care provider’ (10.8%), ‘cooperative leader’ (8.1%), and ‘prestige symbol’ (3.8%) by the first grade medical students – revealing that study participants, in citing such physician- and patient-centered metaphors, may have been reflecting on their own past experiences in the medical system. According to the results, most defined a physician, their aspiring professional identity, as being a significant figure. These images and metaphors of physician have the potential to provide the language of practice for medical students and educators to engage in collaborative dialog to achieve their goals.

Additionally, in analyzing the Turkish context, the findings from this study indirectly highlight the need to look more closely at how medical students perceive themselves in relation to their chosen profession. The associations of students’ status as trainees with these images imply both stability and change in their beliefs of physicians over the course of their physician training. Nonetheless, while changes in first grade medical students’ implicitly held beliefs are desirable and are an expected result of their training, attention must also be given to the direction of curriculum change. Curricular evaluation hinges on measuring whether the goals and objectives of a course have been met by determining whether the desired change in the learner's attitudes, knowledge, or skills has been achieved.

Our findings are limited by several factors. First, despite a substantive sample size, our study was conducted in a single Turkish medical school. Therefore, further research is needed to acquire a more complete picture of how Turkish medical school students (and those in other countries and cultures) think and reason about their professional identity. Future studies may also choose to examine any effects of gender, age, and other grade levels.

Second, this research has focused on a single image: that of a physician. In future studies, perhaps other medical, non-medical (e.g., son, daughter), or social roles (e.g., gender) can be examined relative to students’ identities. Third, the six conceptual themes discussed in this study should be regarded as somewhat speculative, since they were ‘externally devised’ by reviewers rather than generated by the participants themselves). As a result, the dividing lines between some themes may be seen as overly arbitrary. Finally, from the methodology used, it is not clear whether participants provided their ideal images of physicians or an image based on experience. Consequently, future research might ask medical students to focus on and generate two types of metaphors: one to represent an experiential-based image and the other to represent an imagined ideal image. Comparing these two metaphors could provide avenues to understanding the discrepancies between ‘real’ and ‘idealized’ images of physicians in a medical education system.

These limitations notwithstanding, there are several potential implications of these findings on medical curricula. First, medical educators have the responsibility to educate their students to help establish their professional identities. They need, therefore, first-hand information about the candidates to develop a better and deeper understanding of them and to enhance the curriculum. Since metaphors provide simple ways of knowing and seeing the world, medical students’ sense of reality and their own role in their accomplishments will be consistent with these implicit images. These images provide the necessary framework that limits or delimits medical students’ accomplishments of their future health care roles. Being pervasive in the thought and speech of medical students and educators, metaphors can have a profound effect on medical education: they facilitate communication, and give coherence to the distinctive events of a student's medical experience and perception of the profession. Moreover, they can serve as powerful tools for establishing a consensual understanding in the patient-physician relationship and influencing medical experience in ways that promote adaptation and positive self-regard. Although they, like any method, are necessarily limited as a means of describing and formulating a professional identity, they can reflect the personal values and professional identities of participants entering medical school and, subsequently, serve as preliminary ‘fodder’ for curricular revisions.

Second, first grade is a critical stage in medical education. In identifying underlying beliefs at this level, medical students can reflect on their current understanding of the profession to discover points that help or hinder their progress. The roles they consider for themselves, and the perceptions underlying these, can persist over time and, eventually, transform into erroneous beliefs that become resistant to change: metaphor analysis as a reflective tool enables students to recognize and challenge implicit ideas and assumptions that may result in changes to medical education practices. It can also heighten self-awareness, which, in time, leads to informed educational decision making by both students and professors – including ‘thinking outside the box’ of accepted cognitive and situational guidelines (35–37).

Clearly, a future area of related research is to examine whether and how exit level medical students’ conceptual metaphors of physicians change over the educational process. Role perceptions of becoming a physician might change considerably once they enter medical school and/or progress throughout the different stages of becoming a physician (e.g., entry stage, practicum stage, or induction stage). After becoming aware and contemplating their own images, medical students may entertain alternative metaphors for personal consideration. In this way, a process of change could be initiated through awareness of self-chosen images, comparison with alternatives, and the identification of new metaphors more consistent with their own self-images. In the words of Gills and Johnson, metaphors can help us ‘understand the selves we want to become or despair of becoming … the selves we have been and the selves we escaped being … [as well as] the selves we are able to become’. In this worthwhile endeavor, metaphor may be a most potent cognitive device (38).

## References

[1] Coulehan J (2003). Conflicting professional values in medical education. Camb Q Healthc Ethics.

[2] Stern DT, Papadakis M (2006). The developing physician becoming a professional. N Engl J Med.

[3] Association of American Medical Colleges (1999). Teaching professionalism in undergraduate medical education. JAMA.

[4] American Board of Internal Medicine (1995). Project professionalism.

[5] Reisetter M, Korcuska JS, Yexley M, Bonds D, Nikels H, McHenry W (2004). Counselor educators and qualitative research: affirming a research identity. Couns Educ Superv.

[6] Adams K, Hean S, Sturgis P, McLeod Clark J (2006). Investigating the factors influencing professional identity of first year health and social care students. Learn Health Soc Care.

[7] Lingard L, Reznick R, DeVito I, Espin S (2002). Forming professional identities on the health care team: discursive constructions of the ‘other’ in the operating room. Med Educ.

[8] Erikson EH Identity, youth, and crisis.

[9] Niemi H, Stern D, Huber GL (1997). Active learning by teachers. Active learning for students and teachers: reports from eight countries.

[10] Hundert EM, Douglas-Steele D, Bickel J (1996). Context in medical education: the informal ethics curriculum. Med Educ.

[11] Hafferty FW, Franks R (1994). The hidden curriculum: ethics teaching, and the structure of medical education. Acad Med.

[12] Niemi PM (1997). Medical students’ professional identity: self-reflection during the preclinical years. Med Educ.

[13] Gibson DM, Dollarhide CT, Moss J (2010). Professional identity development: a grounded theory of transformational tasks of new counselors. Couns Educ Superv.

[14] Watson C (2006). Narratives of practice and the construction of identity in teaching. Teachers Teach Theor Pract.

[15] Stevens RA (2001). Public roles for the medical profession in the United States: beyond theories of decline and fall. Milbank Q.

[16] Hodgkin P (1985). Medicine is war: and other medical metaphors. Br Med J (Clin Res Ed).

[17] Kanthan R, Mills S (2005). Active learning strategies in undergraduate medical education of pathology: a Saskatoon experience. JIAMSE.

[18] Downing LH, Mujic BK (2009). Infectious diseases are like sleeping monsters: conventional and culturally adapted new metaphors in a corpus of abstracts on immunology. Ibérica.

[19] Danielsson UE, Bengs C, Samuelsson E, Johansson EE (2011). ‘My greatest dream is to be normal’: the impact of gender on the depression narratives of young Swedish men and women. Qual Health Res.

[20] Rofè T (2009). Metaphorical stories for education about mental health challenges and stigma. Schizophr Bull.

[21] Aita V, McIlvain H, Susman J, Crabtree B (2003). Using metaphor as a qualitative analytic approach to understand complexity in primary care research. Qual Health Res.

[22] Fletcher G (2013). Of baby ducklings and clay pots: method and metaphor in HIV prevention. Qual Health Res..

[23] Lakoff G, Johnson M (1980). Metaphors we live by.

[24] Haidet P, Jarecki J, Stuckey H, Teal C, Street RL Using a metaphor to teach communication skills: the ‘jazz and the art of medicine’ course.

[25] Masukume G, Zumla A (2012). Analogies and metaphors in clinical medicine. Clin Med.

[26] Kramsch C, Kalaja P, Barcelos AMF (2003). Metaphor and the subjective construction of beliefs. Beliefs about SLA: new research approaches.

[27] Moser KS (2000). Metaphor analysis in psychology – method, theory, and fields of application. Qual Soc Res.

[28] Saban A, Kocbeker BN, Saban A (2007). Prospective teachers’ conceptions of teaching and learning revealed through metaphor analysis. Learn Instr.

[29] Johnson M (1987). The body in the mind: the bodily basis of meaning, imagination and reason.

[30] Miles MB, Huberman AM (1994). Qualitative data analysis: an expanded sourcebook.

[31] May WF (2001). Beleaguered rulers: the public obligation of the professional.

[32] Rothman DJ, O'Toole T (2013). Ideas for an open society: physicians and the body politic. Occasional Papers from OSI-US Programs, Open Society Foundations Network Web site.

[33] Sullivan WM (2004). Work and integrity: the crisis and promise of professionalism in America.

[34] Bybee RW, Falk JH (2001). Achieving scientific literacy: strategies for insuring that free choice science education complements National formal science education efforts. Free-choice science education: how we learn science outside of school.

[35] Lakoff G, Johnson M (1999). Philosophy in the flesh: the embodied mind and its challenge to Western thought.

[36] Kemp E (1999). Metaphors as a tool for evaluation. Assess Eval High Educ.

[37] MacCorma ER (1990). A cognitive theory of metaphor.

[38] Baake K (2003). Metaphor and Knowledge: The Challenges of Writing Science.

